# Production and Sensory Evaluation of Mixed Spices from Selected Local Spices Retailed in Ede, Nigeria

**DOI:** 10.1155/2023/4404492

**Published:** 2023-02-16

**Authors:** Olufunke Dawodu, Moshood Abibu, Josephine Ajayi, Teslimah Elias

**Affiliations:** Department of Science Laboratory Technology, The Federal Polytechnic Ede, Osun State, Nigeria

## Abstract

Spices are essential group of agricultural products used to enrich taste and enhance nutritional contents of foods and beverages. Spices for various types of food were produced naturally from local available plant materials which have been used since the Middle Ages for flavoring, food additives and preservation, supplement, and/or medicinal purposes. Six spices, namely, *Capsicum annuum* (yellow pepper), *Piper nigrum* (black pepper), *Zingiber officinale* (ginger), *Ocimum gratssimum* (scent leaf), castor seed (ogiri), and *Murraya koenigii* (curry leaf), were selected in their natural forms for the production single spices and mixed spices. These spices were used to determine sensory evaluation on suggested staple food like rice, spaghetti, and Indomie pasta, on a nine-point hedonic scale comprising of taste, texture, aroma, saltiness, mouthfeel, and general acceptability. The results for the sensory evaluation for single and mixed spices were tabulated with value range from least to highest and showed that the mixed spice combinations were preferred to the single spice.

## 1. Introduction

Spices and condiments are essential natural products from dried plants parts used to enrich taste and enhance nutritional contents of foods and beverages for the benefit of human daily life. Spices constitute an important group of horticultural commodities, since antiquity have been considered indispensable in the culinary arts for flavoring foods. Some are used in pharmaceuticals, perfumery, and cosmetics, and several other possess colorant, preservative, antioxidant, antiseptics, and antibiotic properties [1]. Besides, they also play quite a significant role in the national economy of India and also in those of various other spice-producing, exporting, and importing countries of the world [2]. Plant foods used as spices and seasonings are usually aromatic and have pungent dietary taste.

Since the advent of man, herbs and spices have found their usage in not only the improvement of the flavor of edible food [3] but also in the medical aspect in their prevention and treatment of health problems [4, 5]. While there is scientific evidence for the use of such common herbs and medicinal plants, then, had been scarce or lacking, the beneficial aforementioned effects observed from such uses were generally encouraging. It is, therefore, not surprising that the tradition of using such herbs, perhaps even after the advent of modern medicine, has continued [5]. More recently, due to an increased interest in understanding the nutritional effects of herbs/spices more comprehensively [5], several studies have examined the cellular and molecular modes of action of the active chemical components in herbs and their biological properties [6, 7]. Beneficial actions of herbs/spices include anti-inflammatory, antioxidant, antihypertensive, glucoregulatory, and antithrombotic effects. Most of the beneficial effects of spices are usually health-related. One major phytochemical component of herbs and spices is the presence of polyphenol compounds [8, 9]. Some of these healing properties of spices are attributed to the inherent polyphenols in them, and they are often associated with attenuating metabolic syndrome. As time elapses, adverse changes related to these metabolic disorders tend to affect brain and cognition [5]. In addition, the neuroprotective effects of herbs and spices have been demonstrated and, whether directly or indirectly, such beneficial effects may also contribute to an improvement in cognitive function. Spices and herbs have been recognized globally for their dietary and medicinal uses for centuries. A growing body of research is acknowledging their health-promoting properties as well as their therapeutic potential with reference to a number of chronic communicable diseases including cancer and type 2 diabetes [10].

For as long as one could recollect, India is famously called even to date the Land of Spices. They have been able to propagate spices to a very large extent. This propagation has gained international acclaim to the extent that no other country can compete with them in terms of their spice quality and quantity. Even in very small quantities, spice are a welcome sensation to the senses, as they make food more palatable, delicious, and readily assimilated. Most households are familiar with the usage of spices, and the global food processing industry is no stranger to the valuable addition of spices, as this enhances the huge demand for spices which has had continuous rapid increase each passing year [11].

In Nigeria, the use of herbs, spices, and condiments by households and food vendors is quite common and associated with human diet. They are used in our homes for the preparation of food, restaurants, hotels, fast foods, chopping bars, and pizzeria. Information about these spices and condiments are not documented but are passed on by words of mouth over several generations. Most of the commonly essential spices used are imported and are not 100% natural because they have been preserved with lot of chemicals which may affect the body system when consumed. The spice market in Nigeria is so small that it is full of untapped resources; hence, a focal point on local spice production in Nigeria will provide some form of income for the teeming young entrepreneurial population. The aim of this study is to produce acceptable natural spices and seasonings from different locally available plant materials and carry out sensory evaluation on them with stews prepared with them eaten with distinct staple food.

## 2. Materials and Methods

### 2.1. Sample Collection

Six spices, namely, yellow pepper (*Capsicum annuum*), black pepper (*Piper nigrum*), scent leaves (*Occimum gratissimum*), ginger (*Zingiber officinale*), castor seed (“ogiri okpei”), and curry leaf (*Murraya koenigii*), were selected and purchased from the local market in Ede North Local Government, Osun State, Nigeria. The spices were purchased within the periods of October to December 2021. About 2 kg each of the spices were obtained.

### 2.2. Sample Preparation

**Figure 1:**
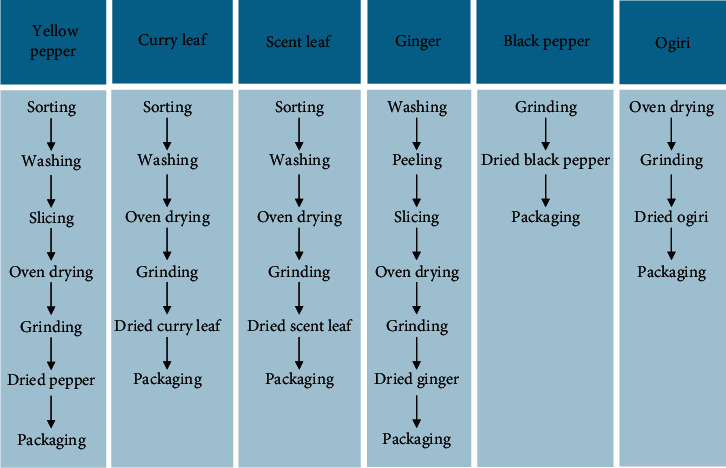
Flow chart on spice preparation.

The purchased spices were prepared accordingly (Figure 1). They were washed, oven-dried at 50°C for 12 hours to remove inherent water molecules, and afterwards, pulverized to form powder. Each powdered sample was placed in an airtight plastic container, labeled, and kept at room temperature until usage.

Mixed spice preparation was as follows: after the spices were in their dried and powdered forms, the mixed spices were prepared according to ratio 1 : 1.

### 2.3. Sensory Evaluation

All spice samples prepared with stews using different kinds of food were evaluated for sensory characteristics on a 9-point hedonic scale [12] by 10 untrained panelists in the Department of Science Laboratory Technology in Federal Polytechnic Ede, Osun State. The 10 panelists (5 males and 5 females) were HND II students of the department between the ages of 20 and 25 years. The 10 untrained panelists were housed in a “nutritional room” made solely for that purpose. The sensory attributes included taste, aroma, texture, flavor, mouthfeel, saltiness, and acceptability. Each panelist was served with food containing different spices. Water was provided to rinse their mouth before evaluating each sample. The panelists were asked to record their observation on a sensory evaluation sheet.

### 2.4. Statistical Analysis

Data are presented as mean ± SE. Spider chart analysis was used for comparison between the spices and the different staple foods used. The sensory data for each attribute were submitted to analysis of the variance (ANOVA), while level of significance (*p* < 0.05) was determined using Bonferroni.

## 3. Results

The present study developed spices from their local plant materials and produced 6 individual spices and 3 mixed spices A, B, and C, making a total of 9 spice preparations in all. From these spices, stews were made with staple food (Indomie, local rice, foreign rice, and spaghetti), and 10 untrained panelists carried out sensory evaluation on them. The sensory evaluation carried out by the trained panelists was based on taste, aroma, texture, flavor, mouthfeel, saltiness, and general acceptability of the spices.

Tables 1 and 2 represent the summary of the findings of the panelists.  Table 1 represents the mean and S.E. of the individual sensory attributes of the single spices, while Table 2 represents the mixed spices.

Using taste as a sensory attribute, from the nine spice preparations, there was a score range of 7.96-8.38, and ginger had the highest score while mixed spice B had the lowest, with majority of the spices having an 8.0 value. The produced spices had significant difference (*p* > 0.05) in taste amongst each other, which showed that the taste of one spice was liked extremely (ginger) and while the other eight were liked moderately (yellow pepper, black pepper, scent leaf, curry leaf, ogiri, and mixed spices A, B, and C) by the panelists.

The next attribute which is aroma had a score range of 7.75-8.25; again, ginger had the highest score while black pepper had the lowest, and the spice mixes A, B, and C had the same scores (7.88) which indicated that all the 9 spices has appreciable aroma.

Texture had score range of 7.67 to 8.13, with curry leaf, black pepper, scent leaf, and mixed spice A having the highest scores, while mixed spice B had the lowest. Although there was significant difference (*p* > 0.05) in the aroma of the spice amongst each other, indicating that the aroma of ginger spice was liked extremely, black pepper was liked slightly while other spices samples (scent leaf, curry leaf, ogiri, yellow pepper, and the mixed spices) were liked moderately by the panelists.

The texture score of the spice samples ranged from 7.67 to 8.13. Curry leaf, scent leaf, black pepper, and mixed spice A had the highest texture scores (8.13) while mixed spice B had the least texture score (7.67). However, the results revealed that there was no significant difference in the texture of the samples, although all spices were liked moderately.

The scores for flavor ranged from 7.75 to 8.38. Ogiri, i.e., castor seed, had the highest texture score (8.38) while mixed spice A had the least score (7.75). However, the results revealed that there was significant difference (*p* > 0.05) in the flavor of the samples, and that all spices were liked extremely.

The mouthfeel score of the spices samples ranged from 7.75 to 8.13. Mixed spice C, scent leaf, black pepper, and ogiri had the highest texture scores (8.13) while mixed spice A had the least mouthfeel score (7.75). However, the result revealed that there was significant difference (*p* > 0.05) in the mouthfeel of the samples, and all were liked moderately.

The saltiness score of the spices samples ranged from 8.00 to 8.38. Curry leaf had the highest texture score (8.38) while mixed spice B had the least score (8.00). However, the result also showed that there was significant difference (*p* > 0.05) in the saltiness of the samples and in the saltiness of each other, which showed that saltiness of curry leaf was liked extremely, ginger was liked moderately while other spices samples (scent leaf, castor seed, black pepper, yellow pepper, and mixed spice B) were liked slightly by the panelists.

The general acceptability of the spices samples ranged from 7.75 to 8.63, mixed spice C had the highest general acceptability score (8.63) followed by curry (8.29), scent leaf black pepper, ogiri, and ginger, and mixed spice A had the least acceptance (8.00). The general acceptability rating implied that the mixed spice C and commercial curry leaf were very much liked than other samples. The general acceptability rating implied that the commercial mixed spice C (comprising of all the spices) was the most liked spice preparation of them all.

There was no spice or spice mix that had the overall best score, but the majority of spices or spice mix had scores above 8.0. No spice or spice combination had less than 7.5 overall score, indicating that the local spices and the spice mix were actually quite good.

**Figure 2:**
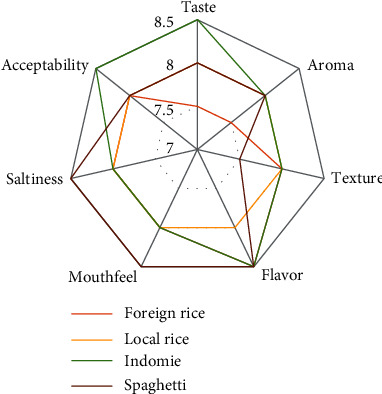
Spider chart analysis of the consumer's preference and acceptability of black pepper.

**Figure 3:**
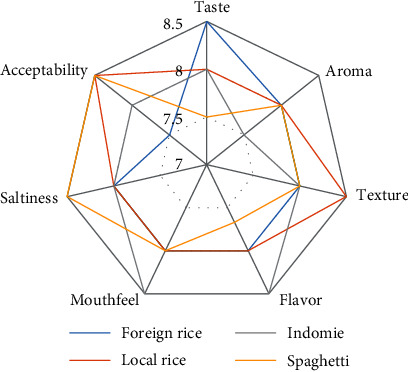
Spider chart analysis of the consumers' preference and acceptability of scent leaf.

**Figure 4:**
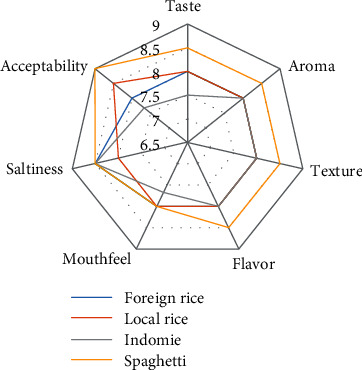
Spider chart analysis of the consumers' preference and acceptability of curry leaf.

**Figure 5:**
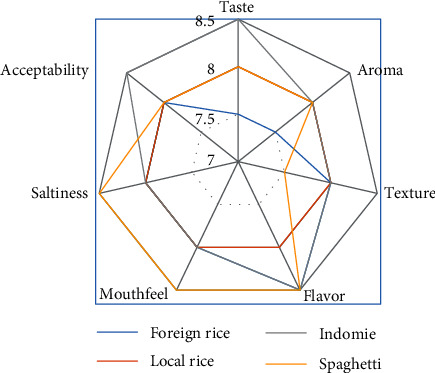
Spider chart analysis of the consumers' preference and acceptability of castor seed.

**Figure 6:**
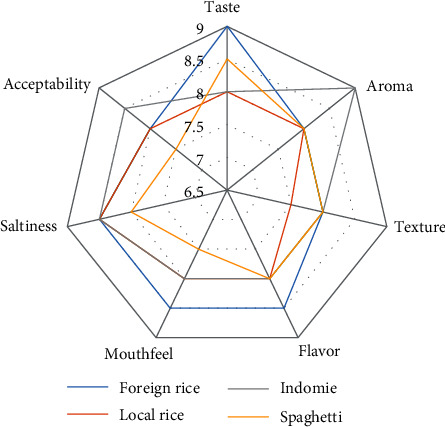
Spider chart analysis of the consumer's preference and acceptability of ginger.

**Figure 7:**
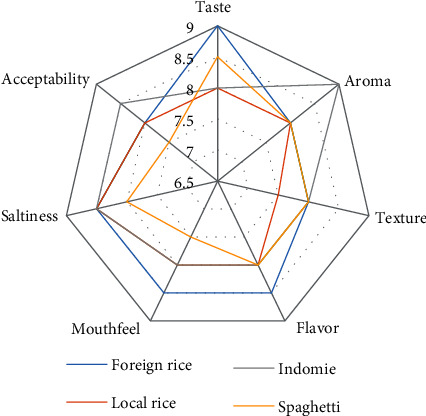
Spider chart analysis of the consumers' preference and acceptability of yellow pepper.

**Figure 8:**
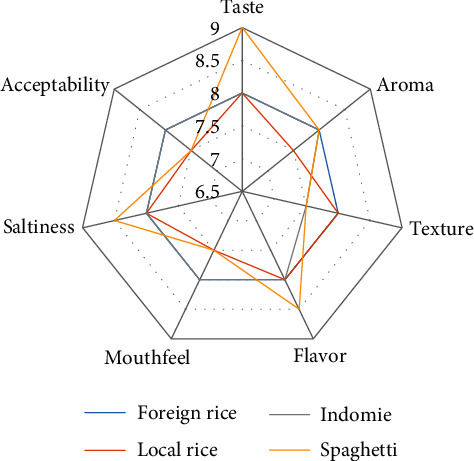
Spider chart analysis of the consumer's preference and acceptability of mixed spice A (yellow pepper, ginger, and curry leaf).

**Figure 9:**
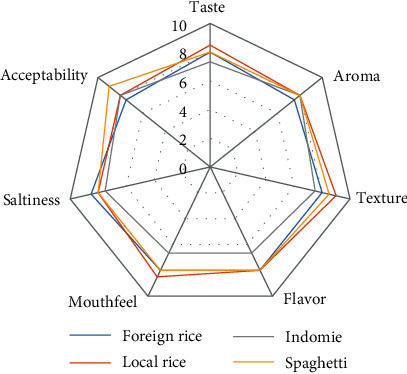
Spider chart analysis of the consumer's preference and acceptability of mixed spice B (black pepper, ogiri, and scent leaves).

**Figure 10:**
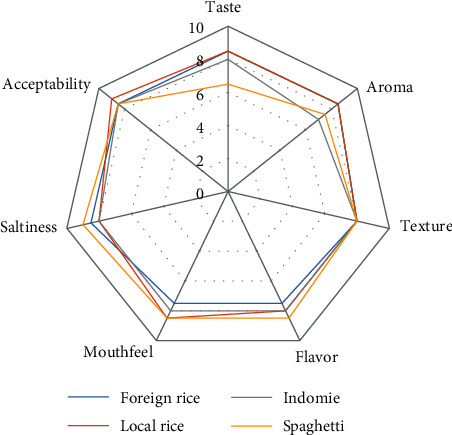
Spider chart analysis of the consumer's preference and acceptability of mixed spice C (yellow pepper, ginger, curry leaves, black pepper, ogiri, and scent leaves).

**Figure 11:**
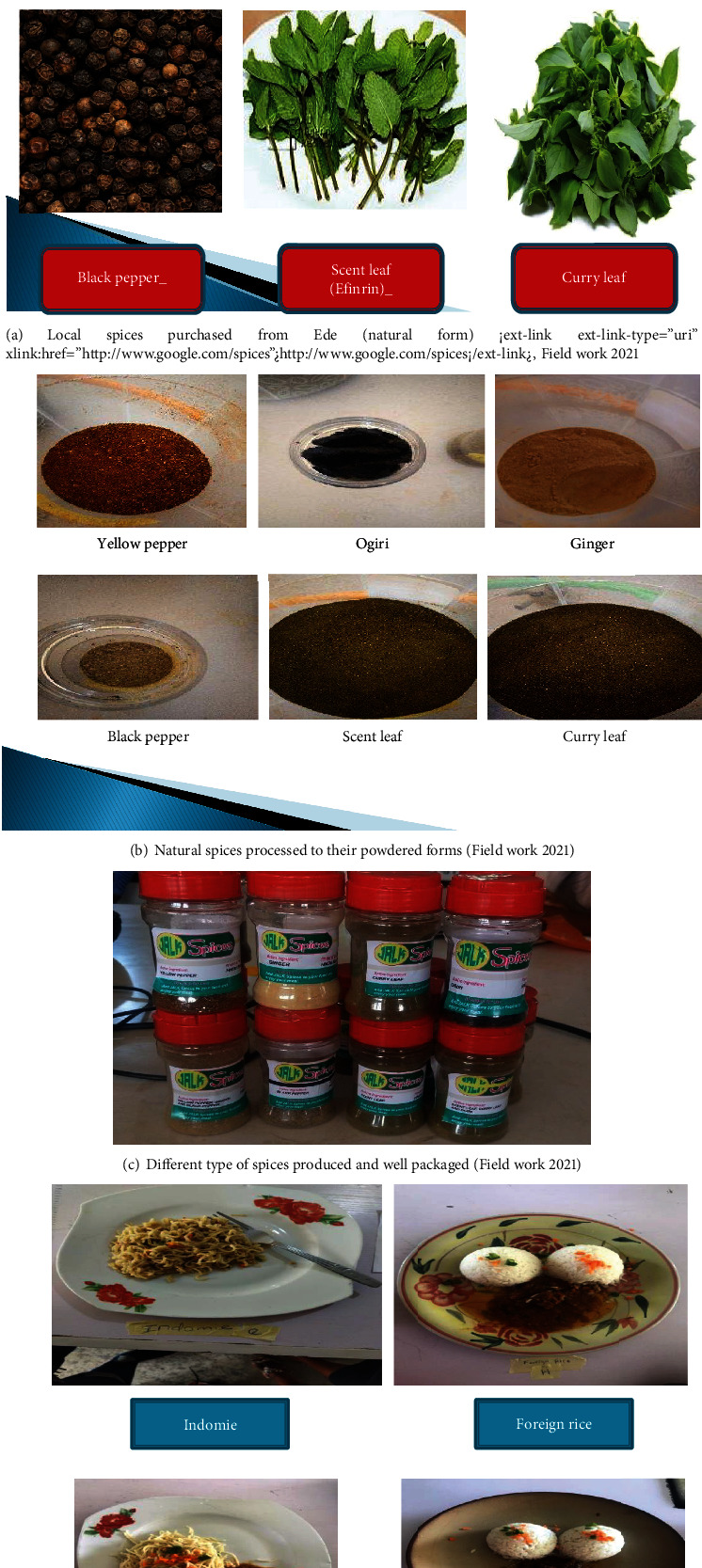
(a-d) Gallery of spices in their local, processed, and packaged forms, with prepared staple food.

Comparison of sensory analysis in spider web charts is given in Figures 2–11. The spider charts actually represent the sensory evaluation of the staple food made from the stews that had the single spices and the spice combinations. Looking at Figures 2–7 that contain the single spices, the sensory attributes varied more, for example, foreign rice had a very good score for taste when used with ginger, scent leaf, and yellow pepper, and such variations existed with the single spices. With the spice mixes A, B, and C, the sensory attributes were generally almost the same with spice mixes B and C having the better scores irrespective of staple food consumed by the panelists, showing a general preference for the spice mixes.

## 4. Discussion

Many studies have evaluated the sensory properties of food in addition to the spice combinations and how they affect food, most studies however are channeled towards specific properties, some for instance are health based, examples include studies checking up on hypertension [13], so as to check the salt content of food, some studies have used spices to improve the taste of beef, pork, and sausages [3], some have looked at the possibility of addition of spices to bread so as to check fungal growth and food packaging [14, 15], and some studies have checked on temperature and the effect of cooking time of different foods. Not many studies, however, have researched on the use of spices with staple food, although a particular study looked at the sensory evaluation of cooking time of different varieties of rice [16]. A particular study looked at the use of e-tongue against human tasting panel in varied tasting sessions [17].

Taste, aroma, texture, flavor, mouthfeel, saltiness, and acceptability are important criteria for evaluating food prepared with different spices. In this study, 10 untrained panelists evaluated food prepared with single spices and mixed spices. The historical use of spices and the long established recipes for food production is of high importance and acceptability. The findings of Aidells and Kelly [18] indicated that spices improve taste, texture, and flavor of food prepared. A study conducted by Abisoye [19] reported that plant phenolic compounds contribute to quality and nutritional value in terms of modifying saltiness, taste, aroma, and flavor; it also provides health beneficial effects.

The essential oil contents in ogiri, i.e., castor seeds, are noted for flavor and taste enhancer [13, 20]; in this study, it did exactly the same thing when it functioned as a single spice and in the spice mixes. Spices add six basic tastes to finished products such as sweet, salty, bitter, sour, spicy, and hot [21]. Spices are not only used for flavoring food but are also used to enhance latent flavor of food [22]. The results from the study showed that all the treatments were almost within the same range. The single spices and the mixed spices added different spice qualities to the staple food. Individual spices are used for specific purposes; sometimes, a particular spice is favored over others, the most use spice in the world is actually salt, and despite its fame, it is usually avoided by hypertensives and not recommended for people with special ailments. In fact, some studies look for salt substitutes so as to help their patients eat well and enjoy their respective dishes. According to the study conducted by Lee [23], spices that have low sodium contents can be used as salt substitute. The present study reveals the correlation between spice and salt in food products. Statistical analysis showed no significant (*p* > 0.05) differences among food prepared with single spices and mixed spices.

## 5. Conclusion

In this study, an attempt was made to develop mixed spices from different single spices (yellow pepper, black pepper, ogiri curry leaf, scent leaf, and ginger). The results for the sensory evaluation showed that the mixed spices were preferred than the single spice due to overall taste. This study has demonstrated that the production of spices from their natural form would improve the taste of the food than the artificial spices, that is, the imported spices.

## Figures and Tables

**Table 1:** Sensory evaluation of single local spices on staple food in Ede.

Spices	Yellow pepper	Ginger	Curry leaf	Scent leaf	Black pepper	Ogiri/castor seed
Taste	8.00 ± 0.41^∗^	8.38 ± 0.48^∗^	8.00 ± 0.41^∗^	8.00 ± 0.41^∗^	8.00 ± 0.82^∗^	8.0 ± 0.41^∗^
Aroma	8.00 ± 0.41^∗^	8.25 ± 0.50^∗^	8.13 ± 0.25^∗^	7.88 ± 0.25^∗^	7.75 ± 0.65^∗^	7.88 ± 0.25^∗^
Texture	7.88 ± 0.25n.s.	7.88 ± 0.25n.s.	8.13 ± 0.25n.s.	8.13 ± 0.25n.s.	8.13 ± 0.48n.s.	7.85 ± 0.25n.s.
Flavor	8.13 ± 0.25^∗^	8.13 ± 0.25^∗^	8.13 ± 0.25^∗^	8.04 ± 0.34^∗^	8.04 ± 0.25^∗^	8.38 ± 0.25^∗^
Mouthfeel	7.88 ± 0.25^∗^	8.00 ± 0.41^∗^	7.92 ± 0.17^∗^	8.13 ± 0.25^∗^	8.13 ± 0.25^∗^	8.13 ± 0.25^∗^
Saltiness	8.13 ± 0.25^∗^	8.34 ± 0.25^∗^	8.38 ± 0.25^∗^	8.13 ± 0.25^∗^	8.13 ± 0.25^∗^	8.13 ± 0.25^∗^
Acceptability	8.25 ± 0.87	8.00 ± 0.41	8.29 ± 0.58	8.1 ± 30.48	8.13 ± 0.82	8.13 ± 0.25

n.s.: no significant difference. ^∗^Significant difference for *p* ≤ 0.05.

**Table 2:** Sensory evaluation of mixed spices on staple food from Ede.

Spices	Mixed spice A	Mixed spice B	Mixed spice C
Taste	8.25 ± 0.50^∗^	7.96 ± 0.48^∗^	7.88 ± 0.95^∗^
Aroma	7.88 ± 0.25n.s.	7.88 ± 0.25n.s.	7.88 ± 0.75n.s.
Flavor	7.75 ± 0.29^∗^	8.25 ± 0.65^∗^	8.00 ± 0.42^∗^
Texture	8.13 ± 0.25^∗^	7.67 ± 0.67^∗^	8.00 ± 0.41^∗^
Mouthfeel	7.75 ± 0.29^∗^	7.98 ± 0.78^∗^	8.13 ± 0.48^∗^
Saltiness	8.13 ± 0.25^∗^	8.00 ± 0.41^∗^	8.38 ± 0.48^∗^
Acceptability	7.75 ± 0.29	8.13 ± 0.63	8.63 ± 0.25

n.s.: no significant difference. ^∗^Significant difference for *p* ≤ 0.05. Key: mixed spices A (yellow pepper, ginger, and curry leaves), B (black pepper, castor seed, and scent leaves), and C (yellow pepper, ginger, curry leaves, black pepper, castor seed, and scent leaves).

## Data Availability

The data used to support the findings of this study are available from the corresponding author upon request.
